# Proximal tibiofibular dislocation associated with fracture of the tibia: a case report

**DOI:** 10.1186/1757-1626-2-196

**Published:** 2009-11-18

**Authors:** Oscar Ares, Xavier Conesa, Roberto Seijas, Lluis Carrera

**Affiliations:** 1 Hospital Quiron, Fundación García Cugat, Plaza Alfonso Comín 5, Barcelona 08023, Spain; 2 PG, Vall D'Hebron 119-129, Traumatologia 2a Planta, Barcelona 08035, Spain

## Abstract

**Introduction:**

We report a case of proximal tibiofibular dislocation associated with an ipsilateral tibial fracture, a rare association of injuries that can remain undiagnosed.

**Case presentation:**

A white 23-year-old man experienced a road accident and was diagnosed with proximal tibiofibular dislocation associated with an open fracture of the tibia and injury to the external popliteus sciatic nerve. He was treated immediately with an intramedullary tibial nail and a cancellous screw at the level of the proximal tibiofibular articulation.

**Conclusion:**

In addition to this case and the surgical treatment, a review of the clinical cases described in the literature is provided, assessing the type of injury and the therapeutic options used, which depend mainly on the stage in which the condition is diagnosed.

## Introduction

Proximal tibiofibular dislocation is an uncommon condition, with 100 cases described up to 1974, when the longest series, which contained 43 patients, was presented [1]. This injury presents as isolated dislocation or chronic instability of the proximal tibiofibular articulation. Association of this condition with other injuries such as tibial fracture is the result of high-energy mechanisms, hence they are usually seen in polytrauma patients. Ogden reported 6 cases of proximal tibiofibular dislocation associated with fracture of the tibia in his series of 43 injuries [1]. However, the number of proximal tibiofibular dislocations associated with tibial fracture in the literature represents only a small percentage of the total of tibiofibular dislocations [2-10].

## Case presentation

A 23-year-old white European Spanish man experienced a motorcycle accident. In the initial examination, the left lower extremity showed deformation and swelling, with multiple injuries and bruises. Distal pulses were palpable and symmetrical. A functional deficit of the extensor muscles of the left foot was noted, raising the suspicion of injury to the external popliteal sciatic nerve.

**Figure 1:**
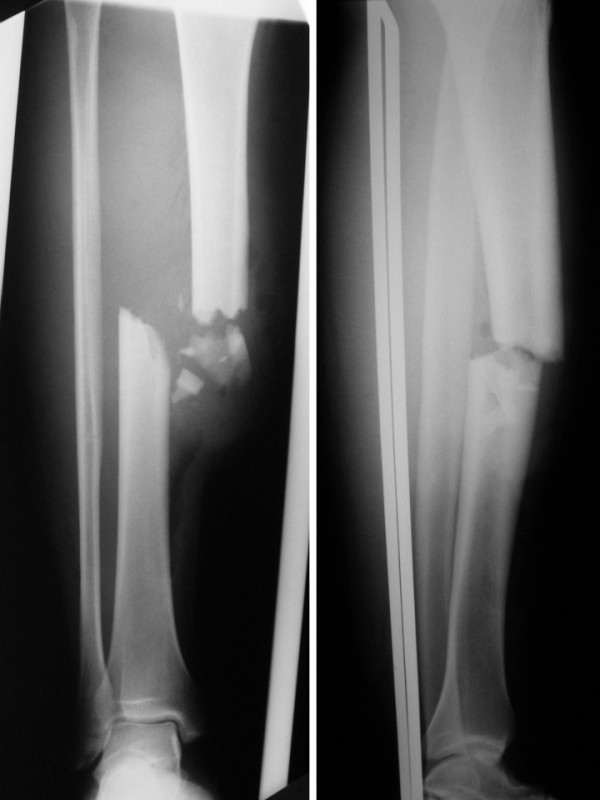
**Anteroposterior and Lateral X-ray**. The open shaft comminuted tibial fracture.

**Figure 2:**
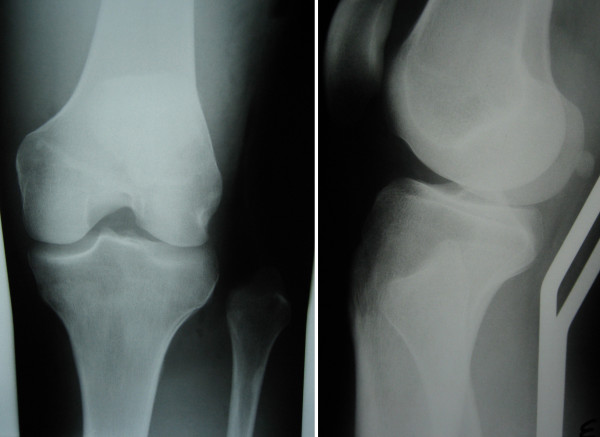
**Anteroposterior and Lateral Knee X-ray**. The proximal tibiofibular dislocation.

The x-ray study confirmed the suspected diagnosis of an open, Gustilo[11] grade IIIA diaphyseal fracture of the tibia (Figure 1), and revealed an associated proximal anterolateral tibiofibular dislocation (Figure 2).

The tibial fracture was treated with an intramedullary nail, performing Friederich debridement of the injuries together with removal of two bone fragments that were displaced from the focus of the fracture. The proximal tibiofibular dislocation was reduced, but due to instability, internal fixation by osteosynthesis with a cancellous screw at the level of the fibular head was carried out to hold reduction. During surgery, we observed an intramural hematoma and lengthening at the level of the external popliteal sciatic nerve. The distal syndesmotic ligaments and the interosseous membrane were not assessed because were stable at the distal part. Only a compressive bandage was used in the postoperative period.

**Figure 3:**
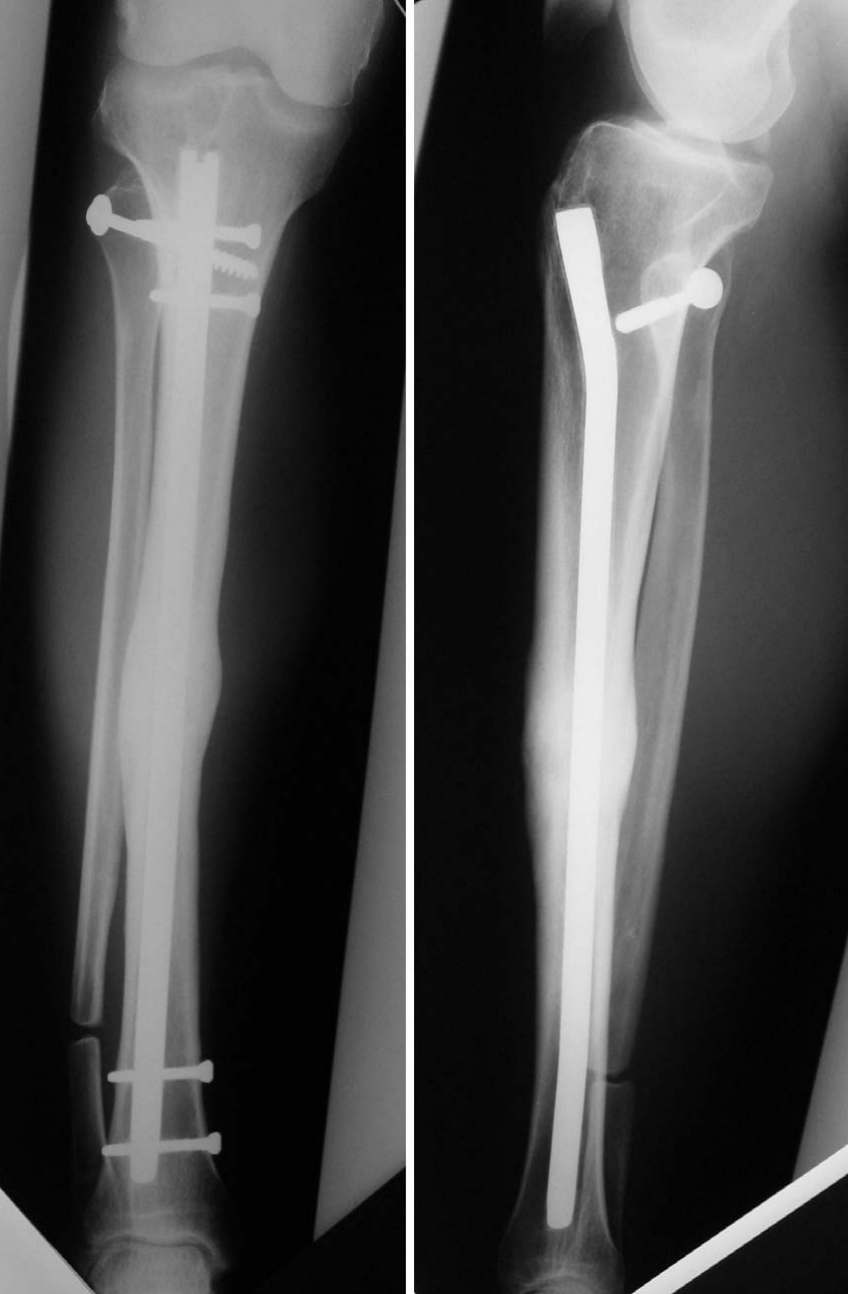
**Anteroposterior and Lateral X-ray**. The open reduction and internal fixation of the tibiofibular dislocation and the intramedullar nail as the treatment of the tibial shaft fracture. The distal fibular osteotomy is done. The consolidation of the fracture is achieved.

During the early postoperative period, a 1.5 to 2 cm diastasis was detected in the tibial fracture, due to fibular length and bone loss. Based on this finding, surgery was again decided at 3 months when no sign of consolidation was observed. This consisted of an osteotomy with resection of 1 cm of fibula, dynamization of the fracture, nail impaction and bone graft contribution (Figure 3). Correct consolidation of the fracture and electromyographic signs of partial popliteal sciatic nerve recovery were observed in subsequent tests.

Two years after the accident, the tibial fracture was completely consolidated, although electromyographic signs of neurological injury persisted at the level of the deep fibular nerve.

## Discussion

The related literature contains only 9 articles describing proximal tibiofibular dislocation associated with tibial fracture, involving a total of 24 cases, with the longest series including 9 such fractures (Table 1)[6]. In most cases, the injury occurred in polytrauma patients who had experienced traffic accidents. The majority were driving a motorbike or had been run over; in two cases the injury was sustained in a car accident. In addition, there was one boat accident, one fall from a horse, and one industrial accident. Only three patients were women[2,7] and there was one child[3]. Patient age ranged from 6 to 55 years, with an average of 24.7 years. Our patient was 23, in keeping with the average age of the cases described.

**Table 1:** Resume of the literature about the several authors of the proximal tibiofibular dislocation.

Authors	Number of cases	Sex	Age	Mechanism	Type of dislocation	EPS Injury	Treatment	EPS Evolution
Brana A.	1	M	20	Boat	superior	No	Orthopedic reduction	

Burgos J	1	M	6	Run over	superior	Yes	Immediate orthopedic reduction	Loss of extension of 5 degrees

Caffinière JY	2	M	22	-	anterolateral	No	Osteotomy	
		
		M	38	-	superior	No	Osteosynthesis, F-T screw	

Dewijze M	2	F	27	Accident at work	anterolateral	No	Abstention	
		
		M	16	Motorbike	posteromedial	Yes	Orthopedic reduction 5 days later	Complete recovery

Gabrion A	9	All M	Media 31	Motorbike	posteromedial	No	Osteosynthesis, F-T screw	
				
				Run over	superior	No	Osteosynthesis, F-T screw	
				
				Motorbike	superior	Yes	Amputation	-
				
				Motorbike	superior	No	Osteosynthesis, F-T screw	
				
				Motorbike	superior	No	Osteosynthesis screw P-T	
				
				Motorbike	inferior	Yes	Amputation	-
				
				Motorbike	inferior	Yes	Amputation	-
				
				Motorbike	inferior	Yes	Amputation	-
				
				Bicycle	inferior	Yes	Amputation	-

Joshi RP	1	F	40	Motorbike	posteromedial	No	Osteosynthesis, F-T screw	

Levy M	1	M	34	Horse	superior	Yes	Removal of the head of the fibula 3 weeks later	Partial recovery

Shelbourne KD	3	M	22	Car	superior	No	Removal of the head of the fibula	
		
		M	24	Car	superior	Yes	Removal of the head of the fibula 16 months later	Complete injury after 2 years
		
		M	18	Run over	superior	No	Osteosynthesis, F-T needles	

Valenti PH	4	M	18	Motorbike	anterolateral	No	Osteosynthesis, F-T screw	
		
		M	23	Motorbike	superior	Yes	Immediate osteosynthesis, F-T screw	Complete recovery
		
		M	18	Motorbike	superior	No	Osteosynthesis, F-T screw	
		
		M	24	Motorbike	superior	Yes	Removal of the head of the fibula 2 months later	Complete recovery

EPS: External politeus sciatic nerve; F-T: fibulotibial

Ogden described four types of proximal tibiofibular dislocation[1]: subdislocation (type I), anterolateral (type II), posteromedial (type III) and superior (type IV). The dislocation patterns associated with tibial fracture include the superior type (14 cases), which always has an associated tibial fracture; the anterolateral type (3 cases), in which isolated tibiofibular dislocation most frequently appears; and the posteromedial type (3 cases). Gabrion[6] described four cases of a type of inferior dislocation produced by high-energy mechanisms; these were associated with tibial fracture and serious vascular injuries, and required urgent amputation.

Proximal tibiofibular dislocation can easily go unnoticed when associated with and ipsilateral tibial fracture in polytrauma patients[1,4,5,8-10,12]. The absence of specific signs and symptoms in the physical examination, the presence of serious associated injuries, and the fact that conventional radiology may not provide useful findings, make a high clinical suspicion together with specific imaging studies, such as computed tomography or comparative x-rays, necessary to establish the diagnosis[1,12,13]. For this reason, some authors believe that the frequency of this entity may be underestimated[4,6].

In 11 out of the 24 cases reported in the literature, an injury associated with the external popliteal sciatic nerve is described. Superior dislocations most often caused popliteal sciatic nerve injury (6 cases), although all the dislocations described as inferior by Gabrion had this associated injury (4 cases)[6]. Our case of anterolateral dislocation is the unique case reporting nerve injury in this type of dislocation.

Patients who completely recovered from the popliteal sciatic nerve injury (4 of the 11 cases with a nerve lesion) received treatment for the tibiofibular dislocation during the first days following the injury (except for one case treated 2 months later), whereas permanent injuries (2 cases) were treated at 3 weeks and 16 months post-trauma. The 5 remaining cases involving popliteal sciatic nerve injury required urgent amputation and the evolution of the neurological lesion could not be assessed. In the case presented herein, the external popliteal sciatic nerve injury occurred in a proximal tibiofibular dislocation of the anterolateral type, the only reported case of this association. The patient was treated urgently for tibiofibular dislocation, but partial injury of the popliteal sciatic nerve persisted two years later.

Treatment for tibiofibular dislocation should be carried out as early as possible to avoid chronic joint instability[4,6,9,10,12]. In cases of dislocation with a tibial fracture, the reduction of the tibial fracture is done first and usually the tibiofibular dislocation is done by itself[2,3,5]. Unstable reduction can be holded with a proximal tibiofibular screw [4,6,7,10,12] or K-wires [9,12]. Other therapeutic options, often used in cases in which the dislocation is diagnosed later, include removal of the fibula head [8-10] or peroneal osteotomy[4]. In the case presented, orthopedic reduction was unstable and osteosynthesis with a proximal tibiofibular screw was carried out. Diastasis of the tibial fracture detected in subsequent tests was treated by osteotomy with removal in the distal third of the fibula. New techniques are now available for treating proximal tibiofibular instability, but their use in patients with this injury and an associated tibial fracture has not been described[12,14-16].

## Conclusion

Proximal tibiofibular dislocation associated with a tibial fracture is an infrequent condition occurring in patients with polytrauma. Because it is rare, this entity can sometimes go unnoticed, delaying the diagnosis. Treatment with Kirschner wires or a temporary tibiofibular screw, carried out during surgery for the tibial fracture, provides good long-term results.

## Consent

Written informed consent was obtained from the patient for publication of this case report and accompanying images. A copy of the written consent is available for review by the Editor-in-Chief of this journal.

## Competing interests

The authors declare that they have no competing interests.

## Authors' contributions

OA performed the clinical follow-up and write the article.

XC performed the bibliographic research and write the article.

RS performed the clinical follow-up and contribute to the write article.

LC performed the emergency and surgical treatment.
